# Prognostic value of adenosine stress perfusion cardiac magnetic resonance with late gadolinium enhancement

**DOI:** 10.1186/1532-429X-13-S1-P117

**Published:** 2011-02-02

**Authors:** Angela G Bertaso, James D Richardson, Dennis Wong, Adam J Nelson, Payman Molaee, Hussam Tayeb, Benjamin K Dundon, Kerry Williams, Matthew I Worthley, Karen S Teo, Stephen G Worthley

**Affiliations:** 1 Cardiovascular Research Centre, Royal Adelaide Hospital & Department of Medicine, University of Adelaide, Adelaide, Australia; 2 Cardiovascular Research Centre, Royal Adelaide Hospital, Adelaide, Australia

## Objective

To determine the prognostic value of a normal adenosine stress perfusion CMR in combination with late gadolinium enhancement in intermediate risk patients with suspected ischaemic heart disease (IHD).

## Background

Adenosine stress CMR is a non invasive test with high sensitivity for the detection of IHD, and is now frequently combined with late gadolinium enhancement (LGE) imaging. This may augment its prognostic accuracy for predicting cardiovascular events. Previous studies have failed to appraise the combination of adenosine stress perfusion CMR with LGE for this purpose, and generally populations at low risk for cardiovascular events have been assessed. We sought to determine the clinical utility of stress perfusion CMR with LGE in the evaluation of intermediate risk patients.

## Methods

Retrospective study of 362 consecutive patients referred to a tertiary cardiology centre for a stress perfusion CMR. Perfusion imaging was obtained at stress (adenosine 140 µg/kg/min) and rest on a 1.5T machine. Late enhancement was assessed with dual pass gadolinium (0.2mmol/kg total dose). A negative test, assessed qualitatively, was defined as the absence of both reversible ischaemia and LGE. Patient records, hospital databases and national death index were reviewed. MACE - death, myocardial infarction, revascularisation or ischaemic hospitalisation - were evaluated over a median follow up of 22 months (IQR 18-25).

## Results

The cohort of 362 patients had a mean age of 62.6 years ±11.9 (mean ±SD), 152 (53.1%) male and 94 (32.8%) had a history of previous MI or PCI. Of the 362, 96 (27%) had a stress perfusion CMR positive for ischaemia, 266 (73%) were negative. Of the 266 negative CMR, MACE was encountered in only 6 (2%) patients (5 PCI and 1 death due to heart failure). Accordingly a negative stress CMR afforded a freedom from MACE of 98%.

## Conclusion

In patients at intermediate risk for cardiovascular events, a negative stress perfusion CMR is associated with an excellent prognosis. This is consistent with the approximate 1% annualised cardiovascular event rate seen in negative stress imaging with other modalities.

